# Cybercrime Victimization and Problematic Social Media Use: Findings from a Nationally Representative Panel Study

**DOI:** 10.1007/s12103-021-09665-2

**Published:** 2021-11-25

**Authors:** Eetu Marttila, Aki Koivula, Pekka Räsänen

**Affiliations:** Economic Sociology, Department of Social Research, University of Turku, Assistentinkatu 7, 20014 Turku, Finland

**Keywords:** Cybercrime, Social media, Problematic social media use, Longitudinal analysis

## Abstract

According to criminological research, online environments create new possibilities for criminal activity and deviant behavior. Problematic social media use (PSMU) is a habitual pattern of excessive use of social media platforms. Past research has suggested that PSMU predicts risky online behavior and negative life outcomes, but the relationship between PSMU and cybercrime victimization is not properly understood. In this study, we use the framework of routine activity theory (RAT) and lifestyle-exposure theory (LET) to examine the relationship between PSMU and cybercrime victimization. We analyze how PSMU is linked to cybercrime victimization experiences. We explore how PSMU predicts cybercrime victimization, especially under those risky circumstances that generally increase the probability of victimization. Our data come from nationally representative surveys, collected in Finland in 2017 and 2019. The results of the between-subjects tests show that problematic PSMU correlates relatively strongly with cybercrime victimization. Within-subjects analysis shows that increased PSMU increases the risk of victimization. Overall, the findings indicate that, along with various confounding factors, PSMU has a notable cumulative effect on victimization. The article concludes with a short summary and discussion of the possible avenues for future research on PSMU and cybercrime victimization.

## Introduction

In criminology, digital environments are generally understood as social spaces which open new possibilities for criminal activity and crime victimization (Yar, 2005). Over the past decade, social media platforms have established themselves as the basic digital infrastructure that governs daily interactions. The rapid and vast adaptation of social media technologies has produced concern about the possible negative effects, but the association between social media use and decreased wellbeing measures appears to be rather weak (Appel et al., 2020; Kross et al., 2020). Accordingly, researchers have proposed that the outcomes of social media use depend on the way platforms are used, and that the negative outcomes are concentrated among those who experience excessive social media use (Kross et al., 2020; Wheatley & Buglass, 2019). Whereas an extensive body of research has focused either on cybercrime victimization or on problematic social media use, few studies have focused explicitly on the link between problematic use and victimization experiences (e.g., Craig et al., 2020; Longobardi et al., 2020).

As per earlier research, the notion of problematic use is linked to excessive and uncontrollable social media usage, which is characterized by compulsive and routinized thoughts and behavior (e.g., Kuss & Griffiths, 2017). The most frequently used social scientific and criminological accounts of risk factors of victimization are based on routine activity theory (RAT) (Cohen & Felson, 1979) and lifestyle-exposure theory (LET) (Hindelang et al., 1978). Although RAT and LET were originally developed to understand how routines and lifestyle patterns may lead to victimization in physical spaces, they have been applied in online environments (e.g., Milani et al., 2020; Räsänen et al., 2016).

As theoretical frameworks, RAT and LET presume that lifestyles and routine activities are embedded in social contexts, which makes it possible to understand behaviors and processes that lead to victimization. The excessive use of social media platforms increases the time spent in digital environments, which, according to lifestyle and routine activities theories, tends to increase the likelihood of ending up in dangerous situations. Therefore, we presume that problematic use is a particularly dangerous pattern of use, which may increase the risk of cybercrime victimization.

In this study, we employ the key elements of RAT and LET to focus on the relationship between problematic social media use and cybercrime victimization. Our data come from high quality, two-wave longitudinal population surveys, which were collected in Finland in 2017 and 2019. First, we examine the cross-sectional relationship between problematic use and victimization experiences at Wave 1, considering the indirect effect of confounding factors. Second, we test for longitudinal effects by investigating whether increased problematic use predicts an increase in victimization experiences at Wave 2.

## Literature Review

### Problematic Social Media Use

Over the last few years, the literature on the psychological, cultural, and social effects of social media has proliferated. Prior research on the topic presents a nuanced view of social media and its consequences (Kross et al., 2020). For instance, several studies have demonstrated that social media use may produce positive outcomes, such as increased life satisfaction, social trust, and political participation (Kim & Kim, 2017; Valenzuela et al., 2009). The positive effects are typically explained to follow from use that satisfy individuals’ socioemotional needs, such as sharing emotions and receiving social support on social media platforms (Pang, 2018; Verduyn et al., 2017).

However, another line of research associates social media use with several negative effects, including higher stress levels, increased anxiety and lower self-esteem (Kross et al., 2020). Negative outcomes, such as depression (Shensa et al., 2017), decreased subjective well-being (Wheatley & Buglass, 2019) and increased loneliness (Meshi et al., 2020), are also commonly described in the research literature. The most common mechanisms that are used to explain negative outcomes of social media use are social comparison and fear of missing out (Kross et al., 2020). In general, it appears that the type of use that does not facilitate interpersonal connection is more detrimental to users’ health and well-being (Clark et al., 2018).

Even though the earlier research on the subject has produced somewhat contradictory results, the researchers generally agree that certain groups of users are at more risk of experiencing negative outcomes of social media use. More specifically, the researchers have pointed out that there is a group of individuals who have difficulty controlling the quantity and intensity of their use of social media platforms (Kuss & Griffiths, 2017). Consequently, new concepts, such as problematic social media use (Bányai et al., 2017) and social networking addiction (Griffiths et al., 2014) have been developed to assess excessive use. In this research, we utilize the concept of problematic social media use (PSMU), which is applied broadly in the literature. In contrast to evidence of social media use in general, PSMU consistently predicts negative outcomes in several domains of life, including decreased subjective well-being (Kross et al., 2013; Wheatley & Buglass, 2019), depression (Hussain & Griffiths, 2018), and loneliness (Marttila et al., 2021).

To our knowledge, few studies have focused explicitly on the relationship between PSMU and cybercrime victimization. One cross-national study of young people found that PSMU is consistently and strongly associated with cyberbullying victimization across countries (Craig et al., 2020) and another one of Spanish adolescents returned similar results (Martínez-Ferrer et al., 2018). Another study of Italian adolescents found that an individual’s number of followers on Instagram was positively associated with experiences of cybervictimization (Longobardi et al., 2020). A clear limitation of the earlier studies is that they focused on adolescents and often dealt with cyberbullying or harassment. Therefore, the results are not straightforwardly generalizable to adult populations or to other forms of cybercrime victimization. Despite this, there are certain basic assumptions about cybercrime victimization that must be considered.

### Cybercrime Victimization, Routine Activity, and Lifestyle-Exposure Theories

In criminology, the notion of cybercrime is used to refer to a variety of illegal activities that are performed in online networks and platforms through computers and other devices (Yar & Steinmetz, 2019). As a concept, cybercrime is employed in different levels of analysis and used to describe a plethora of criminal phenomena, ranging from individual-level victimization to large-scale, society-wide operations (Donalds & Osei-Bryson, 2019). In this study, we define cybercrime as illegal activity and harm to others conducted online, and we focus on self-reported experiences of cybercrime victimization. Therefore, we do not address whether respondents reported an actual crime victimization to the authorities.

In Finland and other European countries, the most common types of cybercrime include slander, hacking, malware, online fraud, and cyberbullying (see Europol, 2019; Meško, 2018). Providing exact estimates of cybercrime victims has been a challenge for previous criminological research, but 1 to 15 percent of the European population is estimated to have experienced some sort of cybercrime victimization (Reep-van den Bergh & Junger, 2018). Similarly, it is difficult to give a precise estimate of the prevalence of social media-related criminal activity. However, as a growing proportion of digital interactions are mediated by social media platforms, we can expect that cybercrime victimization on social media is also increasing. According to previous research, identity theft (Reyns et al., 2011), cyberbullying (Lowry et al., 2016), hate speech (Räsänen et al., 2016), and stalking (Marcum et al., 2017) are all regularly implemented on social media. Most of the preceding studies have focused on cybervictimization of teenagers and young adults, which are considered the most vulnerable population segments (e.g., Hawdon et al., 2017; Keipi et al., 2016).

One of the most frequently used conceptual frameworks to explain victimization is routine activity theory (RAT) (Cohen & Felson, 1979). RAT claims that the everyday routines of social actors place individuals at risk for victimization by exposing them to dangerous people, places, and situations. The theory posits that a crime is more likely to occur when a motivated offender, a suitable target, and a lack of capable guardians converge in space and time (Cohen & Felson, 1979). RAT is similar to lifestyle-exposure theory (LET), which aims to understand the ways in which lifestyle patterns in the social context allow different forms of victimization (Hindelang et al., 1978).

In this study, we build our approach on combining RAT and LET in order to examine risk-enhancing behaviors and characteristics fostered by online environment. Together, these theories take the existence of motivated offenders for granted and therefore do not attempt to explain their involvement in crime. Instead, we concentrate on how routine activities and lifestyle patterns, together with the absence of a capable guardian, affect the probability of victimization.

Numerous studies have investigated the applicability of LET and RAT for cybercrime victimization (e.g., Holt & Bosser, 2008, 2014; Leukfeldt & Yar, 2016; Näsi et al., 2017; Vakhitova et al., 2016, 2019; Yar, 2005). The results indicate that different theoretical concepts are operationalizable to online environments to varying degrees, and that some operationalizations are more helpful than others (Näsi et al., 2017). For example, the concept of risk exposure is considered to be compatible with online victimization, even though earlier studies have shown a high level of variation in how the risk exposure is measured (Vakhitova et al., 2016). By contrast, target attractiveness and lack of guardianship are generally considered to be more difficult to operationalize in the context of technology-mediated victimization (Leukfeldt & Yar, 2016).

In the next section, we will take a closer look at how the key theoretical concepts LET and RAT have been operationalized in earlier studies on cybervictimization. Here, we focus solely on factors that we can address empirically with our data. Each of these have successfully been applied to online environments in prior studies (e.g., Hawdon et al., 2017; Keipi et al., 2016).

### Confounding Elements of Lifestyle and Routine Activities Theories and Cybercrime Victimization

#### Exposure to Risk

The first contextual component of RAT/LET addresses the general likelihood of experiencing risk situations. Risk exposure has typically been measured by the amount of time spent online or the quantity of different online activities – the hours spent online, the number of online accounts, the use of social media services (Hawdon et al., 2017; Vakhitova et al., 2019). The studies that have tested the association have returned mixed results, and it seems that simply the time spent online does not predict increased victimization (e.g., Ngo & Paternoster, 2011; Reyns et al., 2011). On the other hand, the use of social media platforms (Bossler et al., 2012; Räsänen et al., 2016) and the number of accounts in social networks are associated with increased victimization (Reyns et al., 2011).

Regarding the association between the risk of exposure and victimization experiences, previous research has suggested that specific online activities may increase the likelihood of cybervictimization. For example, interaction with other users is associated with increased victimization experiences, whereas passive use may protect from cybervictimization (Holt & Bossler, 2008; Ngo & Paternoster, 2011; Vakhitova et al., 2019). In addition, we assume that especially active social media use, such as connecting with new people, is a risk factor and should be taken into account by measuring the proximity to offenders in social media.

#### Proximity to Offenders

The second contextual component of RAT/LET is closeness to the possible perpetrators. Previously, proximity to offenders was typically measured by the amount of self-disclosure in online environments, such as the number of followers on social media platforms (Vakhitova et al., 2019). Again, earlier studies have returned inconsistent results, and the proximity to offenders has mixed effects on the risk victimization. For example, the number of online friends does not predict increased risk of cybercrime victimization (Näsi et al., 2017; Räsänen et al., 2016; Reyns et al., 2011). By contrast, a high number of social media followers (Longobardi et al., 2020) and online self-disclosures are associated with higher risk of victimization (Vakhitova et al., 2019).

As in the case of risk exposure, different operationalizations of proximity to offenders may predict victimization more strongly than others. For instance, compared to interacting with friends and family, contacting strangers online may be much riskier (Vakhitova et al., 2016). Earlier studies support this notion, and allowing strangers to acquire sensitive information about oneself, as well as frequent contact with strangers on social media, predict increased risk for cybervictimization (Craig et al., 2020; Reyns et al., 2011). Also, compulsive online behavior is associated with a higher probability of meeting strangers online (Gámez-Guadix et al., 2016), and we assume that PSMU use may be associated with victimization indirectly through contacting strangers.

#### Target Attractiveness

The third contextual element of RAT/LET considers the fact that victimization is more likely among those who share certain individual and behavioral traits. Such traits can be seen to increase attractiveness to offenders and thereby increase the likelihood of experiencing risk situations. Earlier studies on cybercrime victimization have utilized a wide selection of measures to operationalize target attractiveness, including gender and ethnic background (Näsi et al., 2017), browsing risky content (Räsänen et al., 2016), financial status (Leukfeldt & Yar, 2016) or relationship status, and sexual orientation (Reyns et al., 2011).

In general, these operationalizations do not seem to predict victimization reliably or effectively. Despite this, we suggest that certain operationalizations of target attractiveness may be valuable. Past research on the different uses of social media has suggested that provocative language or expressions of ideological points of view can increase victimization. More specifically, political activity is a typical behavioral trait that tends to provoke reactions in online discussions (e.g.**,** Lutz & Hoffmann, 2017). In studies of cybervictimization, online political activity is associated with increased victimization (Vakhitova et al., 2019). Recent studies have also emphasized how social media have brought up and even increased political polarization (van Dijk & Hacker, 2018).

In Finland, the main division has been drawn between the supporters of the populist right-wing party, the Finns, and the supporters of the Green League and the Left Alliance (Koiranen et al., 2020). However, it is noteworthy that Finland has a multi-party system based on socioeconomic cleavages represented by traditional parties, such as the Social Democratic Party of Finland, the National Coalition Party, and the Center Party (Koivula et al., 2020). Indeed, previous research has shown that there is relatively little affective polarization in Finland (Wagner, 2021). Therefore, in the Finnish context it is unlikely that individuals would experience large-scale victimization based on their party preference.

#### Lack of Guardianship

The fourth element of RAT/LET assesses the role of social and physical guardianship against harmful activity. The lack of guardianship is assumed to increase victimization, and conversely, the presence of capable guardianship to decrease the likelihood victimization (Yar, 2005). In studies of online activities and routines, different measures of guardianship have rarely acted as predictors of victimization experiences (Leukfeldt & Yar, 2016; Vakhitova et al., 2016).

Regarding social guardianship, measures such as respondents’ digital skills and online risk awareness have been used, but with non-significant results (Leukfeldt & Yar, 2016). On the other hand, past research has indicated that victims of cyber abuse in general are less social than non-victims, which indicates that social networks may protect users from abuse online (Vakhitova et al., 2019). Also, younger users, females, and users with low educational qualifications are assumed to have weaker social guardianship against victimization and therefore are in more vulnerable positions (e.g., Keipi et al., 2016; Pratt & Turanovic, 2016).

In terms of physical guardianship, several technical measures, such as the use of firewalls and virus scanners, have been utilized in past research (Leukfeldt & Yar, 2016). In a general sense, technical security tools function as external settings in online interactions, similar to light, which may increase the identifiability of the aggressor in darkness. Preceding studies, however, have found no significant connection between technical guardianship and victimization (Vakhitova et al., 2016). Consequently, we decided not to address technical guardianship in this study.

Based on the preceding research findings discussed above, we stated the following two hypotheses:

- H1: Increased PSMU associates with increased cybercrime victimization.
- H2: The association between PSMU and cybercrime victimization is confounded by factors assessing exposure to risk, proximity to offenders, target attractiveness, and lack of guardianship.

## Research Design

Our aim was to analyze how problematic use of social media is linked to cybercrime victimization experiences. According to RAT and LET, cybercrime victimization relates to how individuals’ lifestyles expose them to circumstances that increase the probability of victimization (Hindelang et al., 1978) and how individuals behave in different risky environments (Engström, 2020). Our main premise is that PSMU exposes users more frequently to environments that increase the likelihood of victimization experiences.

We constructed our research in two separate stages on the basis of the two-wave panel setting. In the first stage, we approached the relationship between PSMU and cybercrime victimization cross-sectionally by using a large and representative sample of the Finnish population aged 18–74. We also analyzed the extent to which the relationship between PSMU and cybercrime victimization was related to the confounders. In the second stage of analysis, we paid more attention to longitudinal effects and tested for the panel effects, examining changes in cybercrime victimization in relation to changes in PSMU.

### Participants

We utilized two-wave panel data that were derived from the first and second rounds of the Digital Age in Finland survey. The cross-sectional study was based on the first round of the survey, organized in December 2017, for a total of 3,724 Finns. In this sample, two-thirds of the respondents were randomly sampled from the Finnish population register, and one-third were supplemented from a demographically balanced online respondent pool organized by Taloustutkimus Inc. We analyzed social media users (*N* = 2,991), who accounted for 77% of the original data. The data over-represented older citizens, which is why post-stratifying weights were applied to correspond with the official population distribution of Finns aged 18–74 (Sivonen et al., 2019).

To form a longitudinal setting, respondents were asked whether they were willing to participate in the survey a second time about a year after the first data collection. A total of 1,708 participants expressed willingness to participate in the follow-up survey that was conducted 15 months after the first round, in March 2019. A total of 1,134 people participated in the follow-up survey, comprising a response rate of 67% in the second round.

The question form was essentially the same for both rounds of data collection.

The final two-wave data used in the second-stage of analysis mirrored on population characteristics in terms of gender (males 50.8%) and age (M = 49.9, *SD* = 16.2) structures. However, data were unrepresentative in terms of education and employment status when compared to the Finnish population: tertiary level education was achieved by 44.5% of participants and only 50.5% of respondents were employed. The data report published online shows a more detailed description of the data collection and its representativeness (Sivonen et al., 2019).

### Measures

Our dependent variable measured whether the participants had been a target of *cybercrime.* Cybercrime was measured with five dichotomous questions inquiring whether the respondent had personally: 1) been targeted by threat or attack on social media, 2) been falsely accused online, 3) been targeted with hateful or degrading material on the Internet, 4) experienced sexual harassment on social media, and 5) been subjected to account stealing.^1^ In the first round, 159 respondents (14.0%) responded that they had been the victim of cybercrime. In the second round, the number of victimization experiences increased by about 6 percentage points, as 71 respondents had experienced victimization during the observation period.

Our main independent variable was *problematic social media use* (PSMU). Initially, participants’ problematic and excessive social media usage was measured through an adaptation of the *Compulsive Internet Use Scale (CIUS)*, which consists of 14 items ratable on a 5-point Likert scale (Meerkerk et al., 2009). Our measure included five items on a 4-point scale scored from 1 (never) to 4 (daily) based on how often respondents: 1) “Have difficulties with stopping social media use,” 2)”'Have been told by others you should use social media less,” 3) “Have left important work, school or family related things undone due to social media use,” 4) “Use social media to alleviate feeling bad or stress,” and 5) “Plan social media use beforehand.”

For our analysis, all five items were used to create a new three-level variable to assess respondents’ PSMU at different intensity levels. If the respondent was experiencing daily or weekly at least one of the signs of problematic use daily, PSMU was coded as *at least weekly*. Second, if the respondent was experiencing less than weekly at least one of the signs of problematic use, PSMU was coded as *occasionally.* Finally, if the respondent was not experiencing any signs of problematic use, PSMU was coded to none.

To find reliable estimates for the effects of PSMU, we controlled for general *social media use*, including respondents’ activity on social networking sites and instant messenger applications. We combined two items to create a new four-level variable to measure respondents’ social media use (SMU). If a respondent reported using either social media platforms (e.g., Facebook, Twitter), instant messengers (e.g., WhatsApp, Facebook Messenger) or both many hours per day, we coded their activity as *high*. We coded activity as *medium*, if respondents reported using social media *daily*. Third, we coded activity as *low* for those respondents who reported using social media only on a weekly basis. Finally, we considered activity as *very low* if respondents reported using platforms or instant messengers less than weekly.

Confounding variables were related to participants’ target attractiveness, proximity to offenders, and potential guardianship factors.

**Target attractiveness** was measured by *online political activity*. Following previous studies (Koiranen et al., 2020; Koivula et al., 2019), we formed the variable based on four single items: following political discussions, participating in political discussions, sharing political content, and creating political content. Participants’ activity was initially determined by means of a 5-point scale (1 = Never, 2 = Sometimes, 3 = Weekly, 4 = Daily, and 5 = Many times per day). For analysis purposes, we first separated “politically inactive” users, who reported never using social media for political activities. Second, we coded as “followers” participants who only followed but never participated in the political discussions in social media. Third, we classified as “occasional participants” those who *at least sometimes* participated in political activities on social media. Finally, those participants who *at least weekly* used social media to participate in political activities were classified as “active participants.”

**Proximity to offenders** was considered by analyzing *contacting strangers on social media*. Initially, the question asked the extent to which respondents were in contact with strangers on social media, evaluated with a 5-point interval scale, from 1 (*Not at all*) to 5 (*Very much*). For the analysis, we merged response options 1 and 2 to form value 1, and 4 and 5 to form 3. Consequently, we used a three-level variable to measure respondents’ tendency to contact strangers on social media, in which 1 = Low, 2 = Medium, and 3 = High intensity.

**Lack of guardianship** was measured by gender, age, education, and main activity. Respondent’s gender (1 = *Male*, 2 = *Female*), age (in years), level of education, and main activity were measured. While these variables could also be placed under target attractiveness, we placed them here. This is because background characteristics the variables measure are often invisible in online environments and exist only in terms of expressed behavior (e.g., Keipi et al., 2016). For statistical analysis, we classified education and main activity into binary variables. Education was measured with a binary variable that implied whether the respondent had achieved at least tertiary level education or not. The dichotomization can be justified by relatively high educational levels in Finland, where tertiary education is often considered as cut-off point between educated and non-educated citizens (Leinsalu et al., 2020). Main activity was measured with a binary variable that differentiated unemployed respondents from others (working, retirees, and full-time students). Regarding the lack of guardianship, unemployed people are less likely to relate to informal peer-networks occurring at workplaces or educational establishments, a phenomenon that also takes place in many senior citizens’ activities. Descriptive statistics for all measurements are provided in (Table 1).

**Table 1:** Descriptive statistics for the applied variables

Variables	Wave 1			Wave 2		
	n	M	SD	n	M	SD
Cybercrime victimization	2,991			1,134		
No		0.83	0.38		0.80	0.40
Yes		0.17	0.38		0.20	0.40
Problematic social media use (PSMU)	2,701			953		
Never		0.39	0.49		0.37	0.48
Less than weekly		0.33	0.47		0.33	0.47
Weekly		0.20	0.40		0.23	0.42
Daily		0.08	0.27		0.07	0.26
Social media use (SMU)	2,991			1,028		
Very low (ref.)		0.08	0.27		0.05	0.22
Low		0.16	0.37		0.15	0.36
Medium		0.63	0.48		0.66	0.47
High		0.13	0.33		0.14	0.35
Contacting strangers online	2,954			1,130		
Low tendency		0.82	0.38		0.47	0.50
Medium tendency (ref.)		0.13	0.33		0.36	0.48
High tendency		0.05	0.22		0.16	0.37
Online political activity	2,690	0.27	0.44	870	0.24	0.43
Inactive (ref.)		0.30	0.46		0.22	0.42
Follower		0.33	0.47		0.40	0.49
Occasional participant		0.10	0.30		0.14	0.35
Active participant						
Age	2,985	48.85	15.94	1,123	49.85	16.15
Male	2,980	0.48	0.50	1,128	0.50	0.50
Tertiary level education	2,926	0.38	0.49	1,104	0.45	0.50
Unemployed	2,991	0.07	0.24	1,134	0.06	0.23

### Analytic techniques

**Fig. 1:**
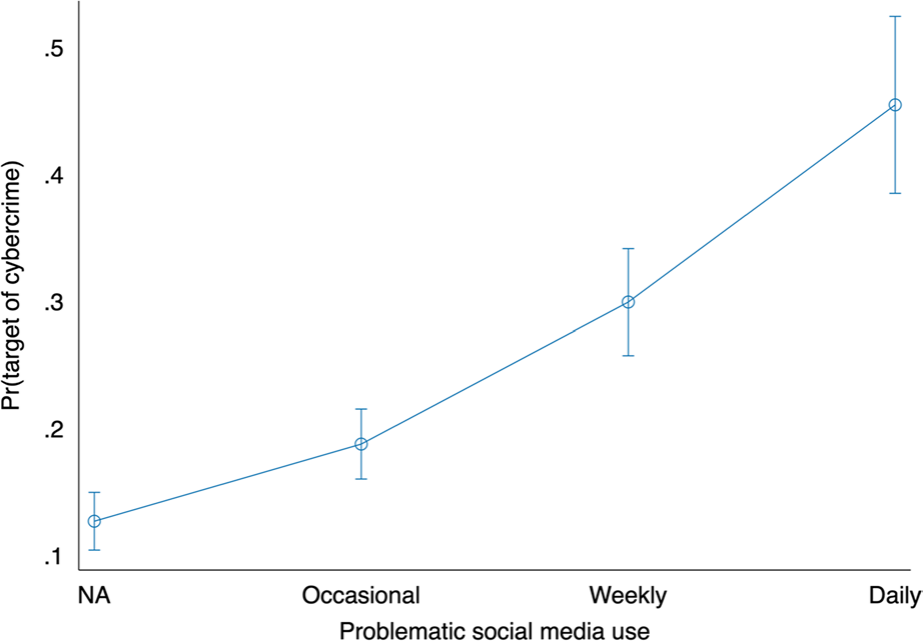
Likelihood of cybercrime victimization according to the level of problematic social media use. Predicted probabilities with 95% confidence intervals

The analyses were performed in two different stages with STATA 16. In the cross-sectional approach we analyzed the direct and indirect associations between PSMU and cybercrime victimization. We reported average marginal effects and their standard errors with statistical significances (Table 2.). The main effect of PSMU was illustrated in Fig. 1 by utilizing a user-written coefplot package (Jann, 2014).

**Table 2:** The likelihood of cybercrime victimization according to confounding and control variables. Average marginal effects (AME) with standard errors estimated from the logit models

Variables	M1	M2	M3	M4	M5
Problematic social media use (PSMU)					
Never (ref.)					
Less than weekly	0.06***	0.05*	0.04*	0.03	0.01
	(0.02)	(0.02)	(0.02)	(0.02)	(0.02)
Weekly	0.17***	0.14***	0.11***	0.09***	0.05*
	(0.02)	(0.02)	(0.02)	(0.02)	(0.02)
Daily	0.33***	0.26***	0.23***	0.21***	0.14***
	(0.04)	(0.04)	(0.04)	(0.04)	(0.04)
Social media use (SMU)					
Very low (ref.)					
Low		0.01	0	-0.01	0
		(0.04)	(0.04)	(0.05)	(0.05)
Medium		0.08*	0.07	0.05	0.02
		(0.04)	(0.04)	(0.05)	(0.05)
High		0.19***	0.15***	0.13**	0.05
		(0.04)	(0.04)	(0.05)	(0.05)
Contacting strangers online					
Medium tendency (ref.)					
Low tendency			-0.11***	-0.08**	-0.07*
			(0.03)	(0.03)	(0.02)
High tendency			0.12*	0.11*	0.10*
			(0.05)	(0.05)	(0.05)
Online political activity					
Inactive (ref.)					
Follower				0	0
				(0.02)	(0.02)
Occasional participant				0.07**	0.07**
				(0.02)	(0.02)
Active participant				0.14***	0.19***
				(0.04)	(0.04)
Age					-0.01***
					(0.001)
Male					-0.04**
					(0.01)
Tertiary					0.001
					(0.03)
Unemployed					0.03
					(0.03)
Observations	2701	2701	2678	2652	2596

Standard errors in parentheses

^***^p < 0.001, **p < 0.01, *p < 0.05

When establishing the indirect effects, we used the *KHB-method* developed by Karlson et al. (2012) and employed the khb command in Stata (Kohler et al., 2011). The KHB method decomposes the total effect of an independent variable into direct and indirect via a confounding / mediating variable (Karlson et al., 2012). Based on decomposition analysis, we reported logit coefficients for the total effect, direct effects, and indirect effects with statistical significances and confounding percentages (Table 3.).

**Table 3:** The decomposition of effect of PSMU on online victimization with respect to confounding factors. The logit coefficients estimated using the KHB method

The effect of PSMU	B	SE		p
Direct	0.65	0.06		< 0.001
Total	0.27	0.06		< 0.001
Indirect	0.38	0.03		< 0.001
The indirect effect of PSMU via:			Confounding %	
SMU	0.03	0.02	7.04	
Contacting strangers	0.07	0.01	17.97	
Political activity	0.12	0.02	31.16	
Age	0.14	0.02	36.13	
Male	0.02	0.01	4.21	
Tertiary	0.00	0.00	-0.1	
Unemployed	0.01	0.01	1.55	

In the second stage, we analyzed the panel effects. We used hybrid mixed models to distinguish two time-varying factors: between-person effects and within-person effects, and predicted changes in cybercrime victimization with respect to changes in problematic social media use. We also tested how the relationship between cybercrime victimization and other time-varying variables changed over the observation period. The hybrid models were performed by using the xthybrid command (Schunck & Perales, 2017).

## Findings

The results for our first hypothesis are presented in Fig. 1. The likelihood of becoming a victim of cybercrime increased significantly as PSMU increased. Respondents who reported problematic use on a daily basis experienced cybercrime with a probability of more than 40%. The probability of becoming a victim was also high, 30%, if problematic use occurred weekly.

The models predicting cybercrime victimization are shown in Table 2. In the first model (M1), PSMU significantly predicted the risk of victimization if a participant reported even occasional problematic use (AME 0.06; *p* < 0.001). If the respondent reported problematic use weekly (AME 0.17; *p* < 0.001) or daily (AME 0.33; *p* < 0.001), his or her probability of becoming a victim was significantly higher.

The next three models (M2-M4) were constructed on the basis of variables measuring risk exposure, proximity to offenders, and target attractiveness. The second model (M2) indicates that highly intensive social media use (AME 0.19, *p* < 0.001) was related to cybercrime victimization. The third (M3) model presents that those who reported low intensity of meeting strangers online had lower probability of being victims (AME -0.11, *p* < 0.001) and those who reported high intensity had higher probability (AME 0.12, *p* < 0.05). Finally, the fourth (M4) model suggests that political activity was related to victimization: those who reported participating occasionally (AME 0.07, *p* < 0.01) and actively (AME 0.14, *p* < 0.001) had higher probability of being a victim.

Next, we evaluated how different guardianship factors were related to victimization. The fifth model (M5) indicates that age, gender, and economic activity were identified as significant protective factors. According to the results, older (AME -0.01, *p* < 0.001) and male (AME -0.04, *p* < 0.001) participants were less likely to be targets of cybercrime. Interestingly, higher education or unemployment was not related to victimization. Finally, the fifth model also suggests that the effect of PSMU remained significant even after controlling for confounding and control variables.

We decomposed the fifth model to determine how different confounding and control variables affected the relationship between PSMU and victimization. The results of the decomposition analysis are shown in Table 3. First, the factors significantly influenced the association between PSMU and victimization (*B* = 0.38, *p* < 0.001), which means that the confounding percentage of background factors was 58.7%. However, the total effect of PSMU remained significant (*B* = 0.27, *p* < 0.001). Age was the most significant factor in the association between PSMU and victimization (*B* = 0.14; *p* < 0.001), explaining 36% of the total confounding percentage. Political activity was also a major contributing factor (*B* = 0.12, *p* < 0.001) that explained 31.2% of the total confounding percentage. The analysis also revealed that meeting strangers online significantly confounded the relationship between PSMU and victimization (*B* = 0.7, *p* < 0.001).

In the second stage, we examined the longitudinal effects of PSMU on cybercrime victimization using panel data from Finnish social media users. We focused on the factors varying in short term, that is why we also analyzed the temporal effects of SMU, contacting strangers online, and online political activity on victimization. The demographic factors that did not change over time or for which temporal variability did not vary across clusters (such as age) were not considered in the second stage.

Table 4 shows the hybrid models predicting each variable separately. The within-effects revealed that increased PSMU increased individuals’ probability of being victimized during the observation period (*B* = 0.77, *p* = 0.02). Moreover, the between-effects of PSMU was significant (*B* = 2.00, *p* < 0.001), indicating that increased PSMU was related to individuals’ higher propensity to be victimized over the observation period.

**Table 4:** Unadjusted logit coefficients of cybercrime victimization according to PSMU and confounding variables from hybrid generalized mixed models

	B	SE	p
Within-subject effects			
PSMU	0.77	0.34	0.02
SMU	0.22	0.66	0.74
Meeting strangers online (Low intensity)	0.58	0.67	0.39
Meeting strangers online (High intensity)	-0.48	0.62	0.44
Online political activity (participation)	0.75	1.23	0.54
Between-subject effects			
PSMU	2.00	0.37	< .0001
SMU	2.00	0.45	< .0001
Contacting strangers online (Low tendency)	-3.27	0.66	< .0001
Contacting strangers online (High tendency)	0.85	0.92	0.36
Online political activity (participation)	2.08	0.59	< .0001

Each variable modelled separately

We could not find significant within-subject effects in terms of other factors. However, the between-effects indicated that SMU (*B* = 2.00, *p* < 0.001), low intensity of meeting strangers online (*B* = -3.27, *p* < 0.001), and online political participation (*B* = 2.08, *p* < 0.001) distinguished the likelihood of individuals being victimized.

## Discussion

Over the last decade, social media has revolutionized the way people communicate and share information. As the everyday lives of individuals are increasingly mediated by social media technologies, some users may experience problems with excessive use. In prior studies, problematic use has been associated with many negative life outcomes, ranging from psychological disorders to economic consequences.

The main objective of this study was to determine whether PSMU is also linked to increased cybercrime victimization. First, we examined how PSMU associates with cybercrime victimization and hypothesized that increased PSMU associates with increased cybercrime victimization (H1). Our findings from the cross-sectional study indicated that PSMU is a notable predictor of victimization. In fact, daily reported problematic use increased the likelihood of cybercrime victimization by more than 30 percentage points. More specifically, the analysis showed that more than 40% of users who reported experiencing problematic use daily reported being victims of cybercrime, while those who never experienced problematic use had a probability of victimization of slightly over 10%.

We also examined how PSMU captures other risk factors contributing to cybercrime victimization. Here, we hypothesized that the association between PSMU and cybercrime victimization is mediated by exposure to risk, proximity to offenders, target attractiveness, and lack of guardianship (H2). The decomposition analysis indicated that confounding factors explained over 50 percent of the total effect of PSMU. A more detailed analysis showed that the association between PSMU and cybercrime victimization was related to respondents’ young age, online political activity, activity to meet strangers online, and intensity of general social media use. This means that PSMU and victimization are linked to similar factors related to routine activities and lifestyle that increase the target's attractiveness, proximity to offenders and lack of guardianship. Notably, the effect of PSMU remained significant even after controlling for the confounding factors.

In the longitudinal analysis, we confirmed the first hypothesis and found that increased PSMU was associated with increased cybercrime victimization in both within- and between-subject analyses. The result indicated a clear link between problematic use and cybercrime experiences during the observation period: as problematic use increases, so does the individual’s likelihood of becoming a victim of cybercrime. At the same time, according to the between-subject analysis, it also appears that cybercrime experiences are generally more likely to increase for those who experience more problematic use. Interestingly, we could not find within-subject effects in terms of other factors. This means, for example, that individuals' increased encounters with strangers or increased online political activity were not directly reflected in the likelihood of becoming a victim during the observation period. The between-subject analyses, however, indicated that an individual’s increased propensity to be victimized is related to higher level of social media activity, intensity of meeting strangers online, and online political activity over time.

Our findings are consistent with those of preceding research pointing to the fact that cybervictimization is indeed a notable threat, especially to those already in vulnerable circumstances (Keipi et al., 2016). The probabilities of cybercrime risk vary in online interactional spaces, depending on the absence and presence of certain key components suggested in our theoretical framework. Despite the seriousness of our findings, recent statistics indicate that cybercrime victimization is still relatively rare in Finland. In 2020, seven percent of Finnish Internet users had experienced online harassment, and 13 percent reported experiencing unwelcome advances during the previous three months (OSF, 2020). However, both forms of cybercrime victimization are clearly more prevalent among younger people and those who use social media frequently.

Cybercrime is becoming an increasingly critical threat as social media use continues to spread throughout segments of the population. Certain online activities and routinized behaviors can be considered to be particularly risky and to increase the probability of cybercrime victimization. In our study, we have identified problematic social media use as a specific behavioral pattern or lifestyle that predicts increased risk of becoming a victim of cybercrime.

Although the overall approach of our study was straightforward, the original theoretical concepts are ambiguously defined and alternative meanings have been given to them. It follows that the empirical operationalization of the concepts was not in line with some studies looking at the premises of RAT and LET framework. Indeed, different empirical measures have been employed to address the basic elements associating with risks of victimization (e.g., Hawdon et al., 2017; Pratt & Turanovic, 2016). In our investigation, we focused on selected online activities and key socio-demographic background factors.

Similarly, we need to be cautious when discussing the implications of our findings. First, our study deals with one country alone, which means that the findings cannot be generalized beyond Finland or beyond the timeline 2017 to 2019. This means that our findings may not be applicable to the highly specific time of the COVID-19 pandemic when online activities have become more versatile than ever before. In addition, although our sample was originally drawn from the national census database, some response bias probably exists in the final samples. Future research should use longitudinal data that better represent, for example, different socio-economic groups. We also acknowledge that we did not control for the effect of offline social relations on the probability of cybercrime risk. Despite these limitations, we believe our study has significance for contemporary cybercrime research.

Our study shows that PSMU heightens the risk of cybercrime victimization. Needless to say, future research should continue to identify specific activities that comprise “dangerous” lifestyles online, which may vary from one population group to another. In online settings, there are a variety of situations and circumstances that are applicable to different forms of cybercrime. For instance, lack of basic online skills regarding cybersecurity can work like PSMU.

In general, our findings contribute to the assumption that online and offline victimization should not necessarily be considered distinct phenomena. Therefore, our theoretical framework, based on RAT and LET, seems highly justified. Our observations contribute to an increasing body of research that demonstrates how routine activities and lifestyle patterns of individuals can be applied to crimes committed in the physical world, as well as to crimes occurring in cyberspace.

## Data Availability

The survey data used in this study will be made available through via Finnish Social Science Data Archive (FSD, http://www.fsd.uta.fi/en/) after the manuscript acceptance. The data are also available from the authors on scholarly request.

## Footnotes

1 1) Have you been targeted by threat or attack on social media? 2) Have you been falsely accused online? 3) Have you been targeted with hateful or degrading material on the Internet? 4) Have you experienced sexual harassment social media? 5) Has your online account been stolen or a new account made with your name without your permission?

## Code Availability

Analyses were run with Stata 16.1. The code is also available from the authors on request for replication purposes.

## References

[CR1] Appel M, Marker C, Gnambs T (2020). Are social media ruining our lives? A review of meta-analytic evidence. Review of General Psychology.

[CR2] Bányai, F., Zsila, Á., Király, O., Maraz, A., Elekes, Z., Griffiths, M. D., et al. (2017). Problematic social media use: Results from a large-scale nationally representative adolescent sample. *PLoS ONE*, *12*(1). 10.1371/journal.pone.0169839 10.1371/journal.pone.0169839 PMC5222338 28068404

[CR3] Bossler AM, Holt TJ, May DC (2012). Predicting online harassment victimization among a juvenile population. Youth & Society.

[CR4] Clark JL, Algoe SB, Green MC (2018). Social network sites and well-being: The role of social connection. Current Directions in Psychological Science.

[CR5] Cohen LE, Felson M (1979). Social change and crime rate trends: A routine activity approach. American Sociological Review.

[CR6] Craig W, Boniel-Nissim M, King N, Walsh SD, Boer M, Donnelly PD (2020). Social media use and cyber-bullying: A cross-national analysis of young people in 42 countries. Journal of Adolescent Health.

[CR7] Donalds C, Osei-Bryson KM (2019). Toward a cybercrime classification ontology: A knowledge-based approach. Computers in Human Behavior.

[CR8] Engström A (2020). Conceptualizing lifestyle and routine activities in the early 21st century: A systematic review of self-report measures in studies on direct-contact offenses in young populations. Crime & Delinquency.

[CR9] Europol (2019). European Union serious and organised crime threat assessment. Online document, available at: https://ec.europa.eu/home-affairs/what-we-do/policies/cybercrime_en

[CR10] Gámez-Guadix M, Borrajo E, Almendros C (2016). Risky online behaviors among adolescents: Longitudinal relations among problematic Internet use, cyberbullying perpetration, and meeting strangers online. Journal of Behavioral Addictions.

[CR11] Griffiths, M. D., Kuss, D. J., & Demetrovics, Z. (2014). Social networking addiction: An overview of preliminary findings. In K. P. Rosenberg & L. C. B. T.-B. A. Feder (Eds.), *Behavioral addictions: Criteria, evidence, and treatment* (pp. 119–141). San Diego: Academic Press. 10.1016/B978-0-12-407724-9.00006-9

[CR12] Hawdon J, Oksanen A, Räsänen P (2017). Exposure to online hate in four nations: A cross-national consideration. Deviant Behavior.

[CR13] Hindelang MJ, Gottfredson MR, Garofalo J (1978). Victims of personal crime: An empirical foundation for a theory of personal victimization.

[CR14] Holt TJ, Bossler AM (2008). Examining the applicability of lifestyle-routine activities theory for cybercrime victimization. Deviant Behavior.

[CR15] Holt TJ, Bossler AM (2014). An assessment of the current state of cybercrime scholarship. Deviant Behavior.

[CR16] Hussain, Z., & Griffiths, M. D. (2018). Problematic social networking site use and comorbid psychiatric disorders: A systematic review of recent large-scale studies. *Frontiers in Psychiatry*, *9*(686). 10.3389/fpsyt.2018.00686 10.3389/fpsyt.2018.00686 PMC6302102 30618866

[CR17] Jann, B. (2014). Plotting regression coefficients and other estimates*. The Stata Journal*, *14*(4), 708–737. 10.1177%2F1536867X1401400402

[CR18] Karlson, K. B., Holm, A., & Breen, R. (2012). Comparing regression coefficients between same-sample nested models using logit and probit: A new method. *Sociological methodology, 42*(1), 286–313. 10.1177%2F0081175012444861

[CR19] Keipi, T., Näsi, M., Oksanen, A., & Räsänen, P. (2016). Online hate and harmful content: Cross-national perspectives. Taylor & Francis. http://library.oapen.org/handle/20.500.12657/22350

[CR20] Kim B, Kim Y (2017). College students’ social media use and communication network heterogeneity: Implications for social capital and subjective well-being. Computers in Human Behavior.

[CR21] Kohler, U., Karlson, K. B., & Holm, A. (2011). Comparing coefficients of nested nonlinear probability models. *The Stata Journal, 11*(3), 420–438. 10.1177/1536867X1101100306

[CR22] Koivula A, Kaakinen M, Oksanen A, Räsänen P (2019). The role of political activity in the formation of online identity bubbles. Policy & Internet.

[CR23] Koivula A, Koiranen I, Saarinen A, Keipi T (2020). Social and ideological representativeness: A comparison of political party members and supporters in Finland after the realignment of major parties. Party Politics.

[CR24] Koiranen I, Koivula A, Saarinen A, Keipi T (2020). Ideological motives, digital divides, and political polarization: How do political party preference and values correspond with the political use of social media?. Telematics and Informatics.

[CR25] Kross E, Verduyn P, Demiralp E, Park J, Lee DS, Lin N (2013). Facebook use predicts declines in subjective well-being in young adults. PLoS ONE.

[CR26] Kross E, Verduyn P, Sheppes G, Costello CK, Jonides J, Ybarra O (2020). Social media and well-being: Pitfalls, progress, and next steps. Trends in Cognitive Sciences.

[CR27] Kuss D, Griffiths M (2017). Social networking sites and addiction: Ten lessons learned. International Journal of Environmental Research and Public Health.

[CR28] Leinsalu M, Baburin A, Jasilionis D, Krumins J, Martikainen P, Stickley A (2020). Economic fluctuations and urban-rural differences in educational inequalities in mortality in the Baltic countries and Finland in 2000–2015: A register-based study. International Journal for Equity in Health.

[CR29] Leukfeldt ER, Yar M (2016). Applying routine activity theory to cybercrime: A theoretical and empirical analysis. Deviant Behavior.

[CR30] Longobardi C, Settanni M, Fabris MA, Marengo D (2020). Follow or be followed: Exploring the links between Instagram popularity, social media addiction, cyber victimization, and subjective happiness in Italian adolescents. Children and Youth Services Review.

[CR31] Lowry PB, Zhang J, Wang C, Siponen M (2016). Why do adults engage in cyberbullying on social media? An integration of online disinhibition and deindividuation effects with the social structure and social learning model. Information Systems Research.

[CR32] Lutz C, Hoffmann CP (2017). The dark side of online participation: Exploring non-, passive and negative participation. Information, Communication & Society.

[CR33] Marcum CD, Higgins GE, Nicholson J (2017). I’m watching you: Cyberstalking behaviors of university students in romantic relationships. American Journal of Criminal Justice.

[CR34] Martínez-Ferrer B, Moreno D, Musitu G (2018). Are adolescents engaged in the problematic use of social networking sites more involved in peer aggression and victimization?. Frontiers in Psychology.

[CR35] Marttila E, Koivula A, Räsänen P (2021). Does excessive social media use decrease subjective well-being? A longitudinal analysis of the relationship between problematic use, loneliness and life satisfaction. Telematics and Informatics.

[CR36] Meerkerk GJ, Van Den Eijnden RJJM, Vermulst AA, Garretsen HFL (2009). The Compulsive Internet Use Scale (CIUS): Some psychometric properties. Cyberpsychology and Behavior.

[CR37] Meshi D, Cotten SR, Bender AR (2020). Problematic social media use and perceived social isolation in older adults: A cross-sectional study. Gerontology.

[CR38] Meško G (2018). On some aspects of cybercrime and cybervictimization. European Journal of Crime, Criminal Law and Criminal Justice.

[CR39] Milani R, Caneppele S, Burkhardt C (2020). Exposure to cyber victimization: Results from a Swiss survey. Deviant Behavior.

[CR40] Näsi M, Räsänen P, Kaakinen M, Keipi T, Oksanen A (2017). Do routine activities help predict young adults’ online harassment: A multi-nation study. Criminology and Criminal Justice.

[CR41] Ngo FT, Paternoster R (2011). Cybercrime victimization: An examination of individual and situational level factors. International Journal of Cyber Criminology.

[CR42] Official Statistics of Finland (OSF) (2020). Väestön tieto- ja viestintätekniikan käyttö [online document]. ISSN=2341–8699. 2020, Liitetaulukko 29. Vihamielisten viestien näkeminen, häirinnän kokeminen ja epäasiallisen lähestymisen kohteeksi joutuminen sosiaalisessa mediassa 2020, %-osuus väestöstä. Helsinki: Tilastokeskus. Available at: http://www.stat.fi/til/sutivi/2020/sutivi_2020_2020-11-10_tau_029_fi.html

[CR43] Pang H (2018). How does time spent on WeChat bolster subjective well-being through social integration and social capital?. Telematics and Informatics.

[CR44] Pratt TC, Turanovic JJ (2016). Lifestyle and routine activity theories revisited: The importance of “risk” to the study of victimization. Victims & Offenders.

[CR45] Reep-van den Bergh CMM, Junger M (2018). Victims of cybercrime in Europe: A review of victim surveys. Crime Science.

[CR46] Reyns BW, Henson B, Fisher BS (2011). Being pursued online. Criminal Justice and Behavior.

[CR47] Räsänen P, Hawdon J, Holkeri E, Keipi T, Näsi M, Oksanen A (2016). Targets of online hate: Examining determinants of victimization among young Finnish Facebook users. Violence and Victims.

[CR48] Schunck, R., & Perales, F. (2017). Within- and between-cluster effects in generalized linear mixed models: A discussion of approaches and the xthybrid command. *The Stata Journal*, 17(1), 89–115. 10.1177%2F1536867X1701700106

[CR49] Shensa A, Escobar-Viera CG, Sidani JE, Bowman ND, Marshal MP, Primack BA (2017). Problematic social media use and depressive symptoms among U.S. young adults: A nationally-representative study. Social Science and Medicine.

[CR50] Sivonen, J., Kuusela, A., Koivula, A., Saarinen, A., & Keipi, T. (2019). *Working papers in economic sociology: Research Report on Finland in the Digital Age Round 2 Panel-survey*. Turku.

[CR51] Wagner M (2021). Affective polarization in multiparty systems. Electoral Studies.

[CR52] Vakhitova ZI, Alston-Knox CL, Reynald DM, Townsley MK, Webster JL (2019). Lifestyles and routine activities: Do they enable different types of cyber abuse?. Computers in Human Behavior.

[CR53] Vakhitova ZI, Reynald DM, Townsley M (2016). Toward the adaptation of routine activity and lifestyle exposure theories to account for cyber abuse victimization. Journal of Contemporary Criminal Justice.

[CR54] Valenzuela S, Park N, Kee KF (2009). Is there social capital in a social network site?: Facebook use and college student’s life satisfaction, trust, and participation. Journal of Computer-Mediated Communication.

[CR55] Van Dijk JA, Hacker KL (2018). Internet and democracy in the network society. Routledge.

[CR56] Verduyn P, Ybarra O, Résibois M, Jonides J, Kross E (2017). Do social network sites enhance or undermine subjective well-being? A critical review. Social Issues and Policy Review.

[CR57] Wheatley D, Buglass SL (2019). Social network engagement and subjective well-being: A life-course perspective. The British Journal of Sociology.

[CR58] Yar M (2005). The novelty of ‘Cybercrime’. European Journal of Criminology.

[CR59] Yar, M., & Steinmetz, K. F. (2019). *Cybercrime and society*. SAGE Publications Limited.

